# Estradiol and social withdrawal in addiction: a sex-specific neuroendocrine perspective

**DOI:** 10.1093/oons/kvag009

**Published:** 2026-08-10

**Authors:** Violet M Kimble

**Affiliations:** Department of Psychiatry, Yale University, 300 George Street, Suite 901, New Haven, CT 06519, United States

**Keywords:** estradiol, addiction, social, withdrawal, intersectionality, relapse

## Abstract

Addiction models have traditionally emphasized drug rewards while often underestimating the role of social processes in relapse vulnerability. Converging evidence from preclinical and clinical studies suggests that social withdrawal during substance use and abstinence may weaken access to social reinforcement, increase perceived stress, and heighten vulnerability to relapse. This brief perspective proposes that estradiol-sensitive signaling shapes, rather than determines, neural systems governing social motivation, stress responsivity, and affective regulation. Across hormonal states, fluctuations or reductions in estradiol signaling may alter the salience of social and stress-related cues, potentially increasing vulnerability to withdrawal-related negative affect. These effects may be further compounded by structural stressors that disproportionately burden Black women, amplifying relapse risk through combined biological and social pathways. Recognizing social withdrawal as a hormonally sensitive and structurally embedded process may help refine addiction models and inform more precise and equitable translational research strategies.

## Introduction

Traditional addiction frameworks conceptualize substance use disorders (SUDs) as conditions driven primarily by maladaptive engagement of the mesolimbic reward circuitry. Drugs of abuse recruit dopaminergic pathways within the ventral tegmental area and nucleus accumbens, progressively biasing behavior toward drug-seeking at the expense of (Sinha et al. 2011) natural reinforcers (Koob and Volkow 2016; Tan et al. 2024). While this reward-centric framework has generated foundational insights, increasing evidence indicates that disruptions in social functioning represent an additional and clinically meaningful dimension of addiction vulnerability.

Social withdrawal is characterized here by reduced social interaction, diminished participation in supportive relationships, or avoidance of social contexts during substance use and abstinence. Importantly, this phenotype should not be interpreted as a unitary marker of reduced affiliative motivation. In clinical contexts, social withdrawal may reflect social anhedonia, shame or stigma surrounding substance use, disrupted relationships, avoidance of substance-associated social networks, reduced access to supportive non-using peers, or the perception that others cannot understand the recovery experience. Both preclinical and clinical studies suggest that social isolation and social stress can increase drug-seeking and relapse vulnerability (Sinha et al. 2011; Mantsch et al. 2016; Havassy et al. 1991). Importantly, social reward and drug reward engage overlapping neural circuitry and may functionally interact (Venniro et al. 2018; Heilig et al. 2016), suggesting that erosion of social reinforcement may remove a protective buffer against relapse. Thus, rather than functioning solely as a downstream consequence of substance use, social withdrawal may contribute to relapse risk, in part through multiple behavioral and social pathways that converge on reduced social engagement.

Sex differences further complicate this vulnerability landscape. Clinically, women report higher rates of stress-induced craving and may exhibit accelerated progression from initial drug exposure to dependence, often described as the telescoping effect (Becker et al. 2017; Greenfield et al. 2010). Preclinical models demonstrate sex differences in stress responsivity, affective regulation, and relapse-like behavior, although the mechanisms underlying these differences remain incompletely understood. Estradiol, the principal circulating ovarian hormone, modulates dopaminergic and stress-sensitive circuits implicated in both reward processing and social behavior (Becker et al. 2017; Diekhof 2018; Calipari et al. 2017; Lynch and Taylor 2005; Bangasser and Valentino 2014; Oyola and Handa 2017; Ycaza Herrera and Mather 2015). However, estradiol should not be equated with female sex. Estradiol signaling occurs across sexes and varies by reproductive status, menstrual or estrous phase, exogenous hormone exposure, age, and endocrine context. Accordingly, sex, gender, and hormonal state should be treated as related but distinct variables in addiction research. Rather than serving as a singular driver of sex differences, estradiol likely functions as a modulatory factor influencing sensitivity to stress, affective state, and social motivation across hormonal contexts.

**Figure 1:**
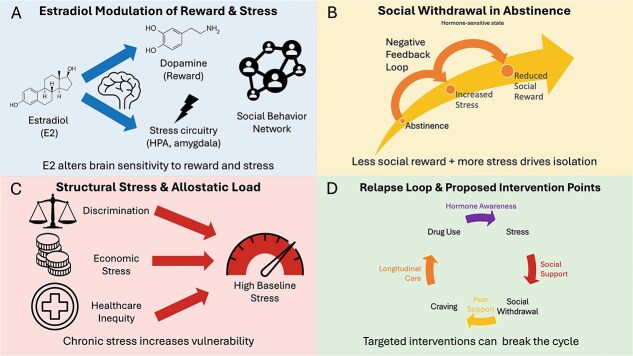
Estradiol-sensitive social withdrawal within a bio-social relapse framework. Panel a illustrates estradiol (E2) modulation of mesolimbic dopamine pathways (VTA–NAc), stress-responsive circuitry (e.g. HPA axis, amygdala), and affective and stress-integrative regions (including the medial prefrontal cortex and lateral habenula). Preclinical evidence indicates that E2 modulates synaptic plasticity and stress responsivity within these circuits in a context- and phase-dependent manner. Panel B depicts social withdrawal during abstinence as a state characterized by reduced engagement with social reinforcers and increased stress sensitivity. Diminished affiliative buffering may heighten negative affect and amplify stress-induced craving, contributing to a reinforcing feedback loop. Panel C overlays structural stress exposure, including chronic discrimination, socioeconomic instability, and healthcare inequities. Persistent stress increases allostatic load and may sensitize stress-responsive neural systems. Because estradiol interacts with these same circuits, hormonal fluctuations may occur against a stress-sensitized neural substrate, particularly in populations experiencing disproportionate structural stress burden. Panel D presents a proposed relapse amplification loop in which stress and social withdrawal reinforce drug-seeking behavior. Potential intervention points include hormone-context-informed monitoring, social reinforcement strategies, peer-support integration, and longitudinal assessment of stress and social functioning. This framework conceptualizes relapse vulnerability as emerging from interacting endocrine, behavioral, and structural processes rather than from reward circuitry alone.

While animal models have been essential for delineating the neural circuitry underlying reward and social behavior, they necessarily simplify the complex social and structural environments shaping human addiction trajectories (Field and Kersbergen 2020). Integrating mechanistic findings from preclinical research with clinical observations of social withdrawal and stress vulnerability may therefore refine existing addiction models. Together, these lines of evidence support a framework in which estradiol-sensitive modulation of reward and stress systems interacts with social behavior and structural context to influence relapse vulnerability (Fig. 1).

## Estradiol and social behavior neurocircuitry

Estradiol (E2), the principal circulating estrogen in premenopausal women, influences neural systems involved in reward, stress integration, and social behavior. Much of the mechanistic evidence describing E2-dependent modulation of these circuits derives from rodent models, which permit circuit-level interrogation of receptor-specific pathways. While these models cannot fully capture the structural and sociocultural complexity of human social experience, they provide critical insight into the neurobiological substrates through which ovarian hormones may influence vulnerability-relevant processes. The goal of this section is not to argue that estradiol uniformly increases or decreases social behavior, but to identify specific circuit-level points at which estradiol signaling can change reward salience, stress sensitivity, and affiliative behavior depending on hormonal state, receptor distribution, and behavioral context.

### Estradiol and dopaminergic reward systems

E2 modulates mesolimbic dopamine signaling within the ventral tegmental area (VTA) and nucleus accumbens (NAc), regions central to reward processing. In rodent models, E2 has been shown to enhance dopamine release and alter dopamine receptor sensitivity in the striatum, influencing motivation and reinforcement learning (Becker et al. 2017; Diekhof 2018; Calipari et al. 2017). These effects are mediated through both genomic and rapid membrane-initiated estrogen receptor signaling. Genomic estrogen receptor signaling can alter transcriptional programs that shape receptor expression, synaptic organization, and longer-timescale changes in neuronal excitability. In contrast, membrane-initiated estrogen receptor signaling can rapidly engage intracellular kinase cascades and local synaptic mechanisms, producing faster changes in dopamine release, synaptic plasticity, and circuit responsiveness (Zhang et al. 2018). These complementary modes of signaling provide a mechanistic basis for how hormonal state may influence reward sensitivity over both acute and longer developmental or reproductive timescales.

Such modulation may contribute to observed sex differences in drug self-administration and reinstatement-like behaviors in animal models (Becker et al. 2017; Lynch and Taylor 2005). Importantly, estradiol’s influence on dopaminergic systems is not limited to drug-related rewards. Because social reward and drug reward engage overlapping mesolimbic circuits (Venniro et al. 2018; Heilig et al. 2016), hormonal modulation of dopamine signaling may plausibly alter the salience of social reinforcement. Rather than uniformly amplifying reward, E2 appears to bias motivational salience in a context-dependent manner, potentially increasing sensitivity to both positive and negative affective stimuli.

### Estradiol, stress pathways, and affective circuits

E2 signaling and stress exposure converge on specific stress- and affect-regulatory nodes, including the hypothalamic–pituitary–adrenal (HPA) axis, amygdala, medial prefrontal cortex (mPFC) and lateral habenula (LHb). This convergence does not by itself establish causality, but it identifies candidate circuits through which hormonal state could shape stress-related affective and avoidance behaviors. Preclinical studies indicate that estradiol interacts with glucocorticoid signaling and modulates stress reactivity, with effects that can vary across hormonal states (Bangasser and Valentino 2014; Oyola and Handa 2017; Ycaza Herrera and Mather 2015). Regions implicated in affective processing and stress integration, including the LHb, amygdala, and mPFC, express estrogen receptors and exhibit hormone-sensitive plasticity (Chen et al. 2019; Lischinsky and Lin 2020; Sato et al. 2020; Siegel and Rosenblatt 1975; Zhang et al. 2018; Ogawa and Parhar 2022; Khetan et al. 2026; Clemens et al. 2019). The LHb, a key node in aversive processing, is strongly engaged by stress and contributes to negative affect and avoidance-related behavior (Zhukovskaya et al. 2024; Shabel et al. 2019).

Hormone-sensitive plasticity may include estrous- or reproductive-state-dependent changes in inhibitory tone, synaptic strength, receptor expression, and functional connectivity within stress- and affect-regulatory circuits (Zhang et al. 2018; Khetan et al. 2026; Clemens et al. 2019). As a result, the same stressor may produce different neural and behavioral consequences depending on hormonal state, with estradiol potentially shaping the magnitude, duration, or recovery of stress-induced adaptations. Chronic stress exposure alters functional connectivity between the amygdala and mPFC and increases LHb activity, neural adaptations associated with negative affect, aversive learning, and stress susceptibility (Zhukovskaya et al. 2024; Shabel et al. 2019; McEwen 2017; McEwen 2007; Koob and Schulkin 2019; Sinha 2008; Liston et al. 2009). Because estradiol modulates synaptic plasticity within these regions, fluctuations in hormonal state may influence the magnitude or persistence of stress-induced neural adaptations. However, evidence also indicates that not all components of social stress reactivity are estrogen-dependent, underscoring that E2 acts as a modulatory rather than a deterministic factor (Trainor et al. 2013).

### Estradiol and social reward/affiliation systems

E2 contributes to social motivation and affiliative behaviors by acting on estrogen receptor–expressing nodes within the social behavior network (Lawal et al. 2025; Brock et al. 2011). In rodent models, estrogen receptor signaling increases oxytocin receptor expression in specific brain regions, providing one mechanism through which hormonal state can alter social recognition and affiliative responsiveness (Champagne et al. 2001; Young et al. 1998). These hormone-sensitive circuits include components of the social behavior network, such as the medial amygdala and hypothalamic nuclei (Chen et al. 2019; Lawal et al. 2025; Bayless et al. 2019; Lee et al. 2014; Lin et al. 2011; Lischinsky et al. 2023). Low E2 states, including ovariectomy or natural cycle phases associated with reduced circulating E2, have been associated in some models with reduced social recognition, altered aggression, or decreased affiliative-related behavior in rodent models (Lawal et al. 2025; Silva et al. 2010; Spiteri et al. 2010). At the same time, social behavior is shaped by multiple interacting neurochemical systems, including endogenous opioids and corticotropin-releasing factor signaling (Heilig and Koob 2007; Machin and Dunbar 2011; Panksepp et al. 1980; Moles et al. 2004; Fabre-Nys et al. 1982; Panksepp et al. 1978), indicating that estradiol operates within a broader neuroendocrine context.

Together, these findings suggest that E2-sensitive modulation of social reward circuitry may influence vulnerability to social withdrawal during periods of abstinence, although direct causal links between estradiol fluctuations and relapse-related social behavior in humans remain to be established.

## Social withdrawal as a mechanistic pathway in addiction

In addiction, social withdrawal may be best understood as a behavioral state that links abstinence-related negative affect, reduced social reinforcement, and stress-sensitive drug seeking. Observable reductions in social contact may arise from multiple pathways, including social anhedonia, avoidance of drug-associated contexts, shame or stigma, disrupted relationships, or reduced access to supportive recovery networks. Regardless of the initiating cause, persistent disengagement can reduce exposure to protective social reinforcement and increase reliance on internal stress-regulation mechanisms already strained during abstinence. Within this framework, social withdrawal is not treated as an isolated cause of relapse, but as a state that can amplify stress, weaken affiliative buffering, and strengthen the coupling between negative affect and drug seeking (Fig. 1).

### Social withdrawal during abstinence/withdrawal

Evidence from rodent models of substance use disorders demonstrates that abstinence from psychostimulants, opioids, and alcohol can reduce social play, decrease social interaction, or increase social avoidance-like behaviors (Achterberg et al. 2014; Pomrenze et al. 2022; Janetsian et al. 2015). Social isolation and social defeat paradigms reliably increase drug-seeking and reinstatement-like responding in animals (Bardo et al. 2013; Miczek et al. 2008; Neisewander et al. 2012; Lu et al. 2003; Bruchas et al. 2010), suggesting that social stress can potentiate relapse-relevant behavior. These findings indicate that social context and affiliative opportunity can increase or decrease drug motivation through circuits involved in reward valuation, stress responsivity, and social reinforcement, although the specific circuit mechanisms differ across substance class, model, and social context.

Parallel observations emerge in human clinical populations. Clinical and recovery-oriented literature describes loneliness, social disconnection, and reduced social engagement as common features of abstinence and recovery contexts (Rokach 2005; Pettersen et al. 2019; Ingram et al. 2020; Kelly et al. 2014; Hosseinbor et al. 2014; Løseth et al. 2025). Social stressors are among the most robust triggers of craving in humans (Sinha et al. 2011; Mantsch et al. 2016), and longitudinal studies demonstrate that supportive social networks predict improved recovery outcomes (Havassy et al. 1991; Kelly et al. 2014). These data support the hypothesis that diminished social reinforcement during abstinence may remove protective regulatory influences over stress and reward systems, thereby increasing relapse vulnerability.

### Behavioral phenotype in estrogen-modulated states

Sex differences in stress responsivity, relapse vulnerability, and social behavior are well documented across preclinical models and clinical observations (Greenfield et al. 2010; Quigley et al. 2021; Becker et al. 2016). Ovarian hormones, including estradiol, have been shown in animal studies to modulate neural excitability and synaptic plasticity, with downstream effects on stress- and reward-related circuits (Diekhof 2018; Calipari et al. 2017; Panksepp et al. 1978). These systems are implicated in both social bonding and social distress.

In rodent models, ovarian hormone depletion has been associated in some paradigms with increased anxiety- or avoidance-like behavior, reduced social investigation, and changes in social recognition or aggression, while estradiol replacement can partially restore specific social or affective behaviors depending on timing, dose, receptor pathway, and assay (Lawal et al. 2025; Silva et al. 2010; Spiteri et al. 2010). However, these findings are not consistent across all models. Stress-induced social withdrawal can occur independently of adult gonadal hormones in some paradigms, and non-estrogenic systems, including opioid, corticotropin-releasing factor, and monoaminergic signaling, also contribute to social withdrawal phenotypes (Trainor et al. 2013; Heilig and Koob 2007; Machin and Dunbar 2011; Panksepp et al. 1980; Moles et al. 2004; Fabre-Nys et al. 1982; Panksepp et al. 1978). Thus, the available evidence suggests that hormonal context may influence the severity, persistence, or recovery of withdrawal-related social disengagement, particularly when abstinence-related stress and reduced social reinforcement occur together.

### Why withdrawal matters for relapse risk

Relapse vulnerability is strongly influenced by stress exposure and affective dysregulation. Stress-induced reinstatement of drug seeking is one of the most robust findings in addiction neuroscience across species (Mantsch et al. 2016). Social rejection, exclusion, and isolation engage neural systems that overlap in part with circuits implicated in craving and affect regulation, including insular, amygdalar, and cingulate regions (Eisenberger 2012; Garavan 2010; Senatorov et al. 2015; Gowin et al. 2014; Maurage et al. 2012; Tomova et al. 2020). Several of these regions, including the insula, amygdala, cingulate cortex, and prefrontal regulatory circuits, are also sensitive to stress exposure and hormonal state (Bangasser and Valentino 2014; Oyola and Handa 2017; Ycaza Herrera and Mather 2015; Zhukovskaya et al. 2024; Shabel et al. 2019; McEwen 2017; McEwen 2007; Koob and Schulkin 2019; Sinha 2008; Liston et al. 2009). This convergence suggests candidate pathways through which social disconnection, negative affect, and craving may become mutually reinforcing, but it does not establish that estradiol directly causes relapse-related social withdrawal in humans.

Within this framework, social withdrawal may amplify relapse risk through multiple pathways. Reduced social engagement can increase perceived stress, diminish access to emotional regulation through supportive relationships, and heighten reliance on substances as a coping mechanism, consistent with attachment-based and self-medication models (Machin and Dunbar 2011; Panksepp et al. 1980; Moles et al. 2004; Fabre-Nys et al. 1982; Panksepp et al. 1978; Khantzian 1997). Rather than operating as an independent driver, social withdrawal may function as a stress-amplifying state that strengthens the coupling between negative affect and drug-seeking behavior. Recognizing this interaction highlights a potential intervention point: restoring social reinforcement and reducing isolation during abstinence may attenuate stress-induced craving and improve recovery trajectories.

## Intersectional Considerations & Equity

The neurobiological mechanisms linking estradiol, stress responsivity, and social withdrawal operate within broader structural contexts that shape exposure to chronic stress and access to care. In the United States, Black women experience disproportionately high levels of cumulative stress exposure due to structural racism, socioeconomic inequities, and healthcare disparities (Forde et al. 2019; Geronimus 1992; Geronimus et al. 2006). Integrating these structural determinants into neuroendocrine models is essential for understanding how biological and social vulnerabilities converge to influence addiction trajectories. In this framework, structural stress is not treated as a distal demographic descriptor but as a chronic exposure that can shape stress physiology, healthcare access, social support, and recovery opportunity.

### Structural stress and the bio-social Cascade

The Weathering Hypothesis proposes that chronic exposure to structural stressors accelerates biological aging and increases allostatic load among Black women (Forde et al. 2019; Geronimus 1992; Geronimus et al. 2006). Persistent activation of stress-response systems, including the HPA axis and sympathetic nervous system, leads to sustained glucocorticoid exposure and altered inflammatory signaling (McEwen 2017; McEwen 2007). Chronic glucocorticoid exposure has been shown to modify neural function and plasticity within stress-sensitive circuits, and converging evidence indicates that such stress-induced neuroadaptations are associated with increased vulnerability to substance use and relapse (Koob and Schulkin 2019; Sinha 2008; Liston et al. 2009).

Estradiol signaling also acts within several stress- and affect-regulatory systems implicated in chronic stress adaptation, including HPA-axis regulation and limbic–prefrontal circuitry (Bangasser and Valentino 2014; Oyola and Handa 2017; Ycaza Herrera and Mather 2015). In preclinical models, E2 modulates these stress-responsive systems, suggesting that hormonal fluctuations could influence how chronic stress is neurobiologically expressed (Bangasser and Valentino 2014; Oyola and Handa 2017; Ycaza Herrera and Mather 2015). When chronic structural stress establishes a heightened baseline of HPA activation, hormonal fluctuations may occur against an already stress-sensitized neural substrate. Under such conditions, reductions in estradiol during abstinence or reproductive transitions could potentially increase stress sensitivity or reduce the stability of social reward processing in vulnerable individuals, a hypothesis that remains to be tested directly. I refer to this interaction as a ‘bio-social vulnerability cascade’: structural stress increases allostatic burden and stress-circuit reactivity; estradiol modulates these same circuits; and social withdrawal during abstinence may be intensified when hormonal (as observed in preclinical models) and structural vulnerabilities (as described in human populations) converge, a hypothesis that warrants direct testing. This framework remains conceptual but is grounded in converging evidence from stress neurobiology and endocrine modulation studies (Fig. 1).

### Importance for the Field

Incorporating structural stress into neuroendocrine models has methodological and translational implications. First, addiction research designs should integrate validated measures of cumulative stress exposure and discrimination alongside hormonal biomarkers, allowing investigators to test whether stress load moderates hormone-related relapse vulnerability. Second, longitudinal studies tracking menstrual phase, reproductive transitions, and social network dynamics could clarify whether hormonally sensitive periods coincide with increased isolation or craving.

Community-engaged research approaches are particularly important in this context. Examples include partnering with community advisory boards to co-design relapse-prevention interventions for Black women, incorporating peer-recovery specialists into study development, embedding digital social-support tools within hormone-informed clinical trials, and adapting intervention materials to reflect participants’ cultural identities, explanatory models of substance use, family and community contexts, and structurally mediated barriers to care (Banks et al. 2023). Existing culturally responsive substance-use frameworks emphasize that such approaches require more than demographic inclusion; they require attention to cultural adaptation, community priorities, language, setting, trust, and access (Banks et al. 2023). Community-driven models such as the Imani Breakthrough project further illustrate how culturally centered recovery supports can be implemented through trusted community institutions, including Black and Latinx churches (Bellamy et al. 2021). By explicitly modeling the interaction between endocrine modulation, social withdrawal, and structural stress exposure, addiction neuroscience can move beyond reductionist frameworks that isolate hormones, drug reward, or individual symptoms from structural stress, gendered experience, social network disruption, and access to culturally responsive care.

## Research & Clinical Implications

Translating the neurobiology of social withdrawal into equitable outcomes requires a fundamental shift in practice. To bridge the gap between mechanistic discovery and clinical reality, the field must operationalize the bio-social insights outlined above. This section outlines a dual-track framework. One track establishes strict estradiol-aware experimental standards to strengthen reproducibility. The other applies precision clinical approaches that address the hormonal and structural mechanisms linked to relapse.

### Estradiol-aware experimental standards

Advancing this framework requires greater rigor in how sex, gender, and hormonal status are operationalized in addiction research. Although ovarian hormones are known to influence stress responsivity and drug-related behaviors, estrous cycle phase and broader ovarian hormone context are inconsistently reported in both preclinical and clinical studies (Arunogiri et al. 2021; Vázquez-Morales et al. 2025). Transparent reporting of biological sex, self-identified gender, reproductive status, and hormone exposure would improve reproducibility and clarify the conditions under which relapse vulnerability may vary. These reporting practices would also reduce the risk of treating estradiol as a proxy for female sex, because they separate biological sex, self-identified gender, reproductive status, and measured or inferred hormone exposure.

In preclinical models, detailed reporting of estrous stage and ovariectomy parameters should become standard practice when studying stress or social behavior. In human research, integrating menstrual phase tracking, contraceptive status, and reproductive transitions into study design would allow investigators to test whether relapse-related outcomes fluctuate across hormonal contexts. Importantly, sex and gender should be treated as related but distinct variables, particularly when studying social withdrawal, which is shaped by both biological modulation and gendered social experience. In parallel, addiction paradigms should expand outcome measures to routinely include validated assessments of social functioning, perceived isolation, and social network structure. Incorporating these measures would permit direct testing of whether hormone-sensitive periods coincide with measurable changes in social engagement or stress reactivity.

### Translational Pathways & Precision Medicine

Clinically, this framework suggests that relapse prevention strategies may benefit from incorporating reproductive hormone context and social vulnerability markers into risk stratification models. Rather than advocating for immediate hormone-based interventions, a first step is observational: prospective studies could examine whether craving intensity, social withdrawal, or relapse events cluster within specific menstrual phases, perimenopausal transitions, or postpartum periods. If such associations are observed, adaptive care models could be developed in which social-support interventions are intensified during hormonally sensitive windows. For example, peer-recovery engagement, structured social reinforcement programs, or digital social-support tools could be front-loaded during periods associated with increased stress sensitivity. For structurally marginalized populations, these adaptive care models should be developed with community partners so that intensified support during hormonally sensitive windows is accessible, culturally responsive, and not framed as an individual biological deficit.

More broadly, the concept of ‘precision addiction medicine’ in this context refers to integrating endocrine biomarkers, validated measures of cumulative stress exposure, and social network metrics into predictive models of relapse vulnerability. Such models would require longitudinal data collection and rigorous validation before clinical deployment. By grounding precision approaches in measurable biological and structural variables, this framework aims to bridge mechanistic neuroscience with equitable care delivery rather than proposing hormone manipulation as a standalone solution.

## Conclusion

Addiction science has historically been dominated by drug reward circuitry, but growing evidence indicates that stress, social disconnection, and hormonal context also shape relapse vulnerability. This perspective argues that estradiol-sensitive signaling may contribute to individual differences in stress responsivity, social motivation, and affective regulation without acting as a deterministic cause of addiction or social withdrawal. Social withdrawal should therefore be studied as a heterogeneous and clinically meaningful state that may amplify relapse risk by weakening social reinforcement and increasing negative affect during abstinence. Integrating sex, gender, hormonal state, and structural stress into addiction models provides a framework for advancing precise and individualized care.

## Supplementary Material

Supplementary_file_for_export_kvag009

## Data Availability

No new data were generated or analyzed in support of this research.
