# Supplementary material for: Estradiol and social withdrawal in addiction: a sex-specific neuroendocrine perspective
Source: Oxf Open Neurosci. 2026 Aug 10;5:kvag009. doi: 10.1093/oons/kvag009 (PMC13464682; doi:10.1093/oons/kvag009)
Supplement: Supplementary file 1 [file supplementary_file_for_export_kvag009.docx]

**Round 1**

01-Feb-2026

Dear Dr. Kimble,

Manuscript ID OXFNSC-2025-008 entitled "Estradiol and Social Withdrawal in Addiction: A Sex-Specific Neuroendocrine Perspective" which you submitted to the Oxford Open Neuroscience, has been reviewed. The comments of the reviewer(s) are included at the bottom of this letter.

Please note that this journal operates with transparent peer review. This means that if your submission is accepted for publication, the full peer review history of your article will publish online alongside your article. This includes reviewer comments, editor decision letters, and your author responses.

The reviewers suggest some major revisions to your manuscript. Therefore, I invite you to respond to the reviewers' comments and revise your manuscript within the next 60 days from today date. In addition to their suggestions about more carefully including supporting citations and literature, please provide a visual illustrating the main concepts and themes of your perspective. This will help the reader with the take-home message.

To revise your manuscript, log into https://mc.manuscriptcentral.com/oxfnsc and enter your Author Centre, where you will find your manuscript title listed under "Manuscripts with Decisions."  Under "Actions," click on "Create a Revision."  Your manuscript number has been appended to denote a revision.

You may also click the below link to start the revision process (or continue the process if you have already started your revision) for your manuscript. If you use the below link you will not be required to login to ScholarOne Manuscripts.

*** PLEASE NOTE: This is a two-step process. After clicking on the link, you will be directed to a webpage to confirm. ***

https://mc.manuscriptcentral.com/oxfnsc?URL_MASK=0356ca8b0e314e278630963e2b73d096

You will be unable to make your revisions on the originally submitted version of the manuscript.  Instead, revise your manuscript using a word processing program and save it on your computer.  Please also highlight the changes to your manuscript within the document by using the track changes mode in MS Word or by using bold or colored text.

Once the revised manuscript is prepared, you can upload it and submit it through your Author Centre.

When submitting your revised manuscript, you will be able to respond to the comments made by the reviewer(s) in the space provided.  You can use this space to document any changes you make to the original manuscript.  In order to expedite the processing of the revised manuscript, please be as specific as possible in your response to the reviewer(s).

IMPORTANT:  Your original files are available to you when you upload your revised manuscript.  Please delete any redundant files before completing the submission.

Because we are trying to facilitate timely publication of manuscripts submitted to the Oxford Open Neuroscience, your revised manuscript should be uploaded within the next 60 days from today date.  If it is not possible for you to submit your revision in a reasonable amount of time, we may have to consider your paper as a new submission.

Once again, thank you for submitting your manuscript to the Oxford Open Neuroscience and I look forward to receiving your revision.

Sincerely,
Prof. Alicia Izquierdo
Senior Editor, Oxford Open Neuroscience
aizquie@psych.ucla.edu


Reviewer: 1

Comments to the Author
In this perspective, entitled “Estradiol and Social Withdrawal in Addiction: A Sex-Specific Neuroendocrine Perspective, authors provide an interesting and novel series of connections between addiction, social withdrawal, estradiol, and Black populations. Although this perspective is important and worthy of exploration, the key arguments of the perspective are, at best, loosely connected. More concerning however is the use of references that do not support the statements claimed in the paper. As such, although the paper has merit and warrants further investigation, it is currently not suitable for publication until the arguments posited are better connected and supported by appropriate references. Finally, future directions need to be better explained for the novelty of the work to shine.

Major concerns:
In the abstract, authors state multiple arguments that they set out to defend in the paper: 1) Social withdrawal is not just a consequence but a driver of relapse risk, 2) estradiol is a key biological regulator of social motivation/withdrawal, and 3) Estradiol-linked social effects are compounded by structural stressors that disproportionately burden marginalized populations.
The first argument regarding social withdrawal is perhaps the least novel, and accordingly has the most scientific support. Still, the references are not cited appropriately here. For example, “Social reward deficits function not merely as a consequence of drug use, but as a potentiating factor in addictive behavior” cites Venniro et al.’s seminal paper demonstrating that drug-exposed rats will prefer to operantly behave for a social reward over a drug reward. This paper does not support the notion that social reward deficits are a consequence of drug use and does not support the notion that social reward deficits are a potentiating factor in addictive behavior. This is one of many examples throughout the manuscript where a reference was selected without a close read of the findings.
Regarding the second argument, authors are forceful in their linkage of estradiol to social motivation and reward. Although estradiol may be linked to social motivation, softening of this language may be appropriate for this perspective given the limited knowledge of the field. For example, authors claim that “…females also manifest heightened sensitivity to negative affective states during abstinence compared to males. Neuroendocrine factors, specifically 17B-estradiol, largely drive these sex-specific differences.” They then cite an influential review by Becker et al., which does indeed claim that females may have negative affective states during abstinence, but does not link this to estradiol and in fact, mainly uses animal studies when discussing a potential role of estradiol.
- Another example of this is found in section 3.2, where authors state “the manifestation of social withdrawal is distinctly reliant on neuroendocrine states” without a citation. They then state that stress responsivity, relapse vulnerability, and social interaction have sex differences, citing a 2010 Cahill review which focuses on emotional memory: “even stating sex hormones are crucial to the enhanced detail memory for emotional events in women, and in ways that remain almost wholly unexplored.” Given this citation, the claim that social withdrawal is reliant on neuroendocrine states, while likely true, is undersupported here.
- Further, limitations of the arugment are not discussed here. For example, although Brian Trainor, a pioneer in the field, has been cited, he has demonstrated that elements of social withdrawal are not estrogen-dependent (see Trainor et al., 2013, Hormones and Behavior). A limitations section would be balanced an appropriate.
For the third argument, although critically necessary for future lines of research, it is perhaps not appropriate to state that in the abstract that the perspective will be discussing marginalized populations more generally when the discussion is specifically about Black individuals and Black women. One concern about stating “marginalized populations” is at worst, it can do a disservice to a specific marginalized population by suggesting that mechanisms of subjugation are generalized.
Here, authors are discussing structural racism and state “When these factors interface with hormonal vulnerabilities, they amplify susceptibility to SUDs and create pronounced inequities in access to comprehensive addiction care and support,” citing a 2011 paper by Algeria et al.. This paper does not discuss hormones or hormonal vulnerabilities, and so data would be necessary to make the likely accurate, but here unsupported, claim that systemic racism compounds with hormonal vulnerabilities.
Other concerns
“animal models… often fail to capture the complexity of human clinical social presentations” is stated early in the manuscript, yet animals studies are quoted for the bulk of the perspective, typically without naming the model. Although this is true that animal models have limitations, their utility is not well described, and their contributions to the field are obfuscated. For example, in the section “Estradiol and Social Behavior Neurocircuitry,” every behavioral reference in this section refers to animal studies (refs 14-23) and yet rodents are not mentioned once. Throughout the manuscript, findings are often discussed without naming the model, and rodent and human studies are interspersed in a way that can be confusing or misleading.


Reviewer: 2

Comments to the Author
In this Brief Perspective, the authors present a relationship between the hormonal and societal influences on social withdrawal and affective neural circuitry driving drug addiction and relapse. This effort to bridge evidence from basic science studies and social epidemiology could be an important contribution to current conversations surrounding the need to better understand and address social determinants of health, both in clinical settings and when designing translational research experiments. The discussion of chronic stress and intersectional challenges faced by marginalized communities also brings up a factor that is inherently absent from preclinical studies but extremely relevant to the treatment of substance use conditions: the structural influences that many people experiencing addiction face, which can significantly impact a person’s ability to access, engage with, and maintain treatments. Overall, the manuscript provides a compelling conceptual bridge between neuroendocrine signaling and structural social determinants in addiction. However, the current draft suffers from significant citation inaccuracies and conceptual gaps that must be addressed before publication. Specifically, the linking evidence between these claims leaves much to be desired. Throughout the article, there are broad claims that lack sufficiently specific evidence. Linking mechanisms need more evidence, and actionable suggestions should be bolstered by direct steps towards implementation. While this perspective begins an important conversation, major revisions are necessary to make this suitable for publication. With revisions that more thoroughly and accurately cite evidence, this has the potential to add a valuable discussion of intersectional translational research in the field.

Major Revisions
Sources need to be thoroughly reviewed. Several citations did not seem to fit the claim. To name a few:
- Section 2.1’s statement on dopamine synthesis, release and uptake inhibition cites [25] (Luine and Frankfurt, Neuroscience 2013), which doesn’t mention anything about dopamine. This source appears to be cited multiple other times in which it may not be appropriate, including section 2.3’s discussion of oxytocin.
- Section 2.3 claim about E2 strengthening the rewarding value of social interactions through increasing OTR expression also cites [38] (Trainor et al, Hormones and Behavior 2008), which is about photoperiod and doesn’t mention oxytocin or reward.
- Section 4 introduces the Weathering Hypothesis and discussion of intersectional structural considerations extremely vaguely and makes little attempt to provide evidence for the “bio-social vulnerability cascade.”

Section 4 could be improved by further discussion of community-engaged research methodologies: provide examples of successful studies or how current models could be improved, rather than just stating that these should be implemented. Also, provide specific evidence for the mechanistic cascade; i.e., how exactly does high allostatic load interact with E2-sensitive circuits like the mPFC or LHb to accelerate the loop?

Similarly, Section 5 introduces “precision addiction medicine” and “targeted hormone modulation” within one brief paragraph, overall contains limited sources, and does not seem sufficient to convince a clinical or basic science audience that these methodologies would be effective. Suggest that authors provide concrete examples of community-engaged research methodologies rather than just stating they should be used.

Source [95] (Yamac and Bakir, Rev Assoc Med Bras 1992; 2025) should also be reviewed, as it is used in a statement about targeted hormone modulation but seems irrelevant.


Minor Revisions

Section 3.1 clearly delineates evidence from rodent SUD models versus human clinical evidence. This approach should be taken more frequently, because in other sections it was at times unclear whether behaviors/neuroendocrine patterns described human or animal studies.  

In line 14-15 the use of 4“cruel” to describe this paradoxical phenomenon introduces a personal tone that feels less objective than the rest of the perspective.

The recommendation for "mandatory reporting standards" in Section 5.1 is a strength but should explicitly mention the importance of reporting both sex and gender variables to align with the intersectional goals of the paper.

Associate Editor
Comments to the Author:
Both reviews support the importance of this perspective and its inclusion in the literature. However, referencing and clear support of particular arguments is to be largely revised among other key areas (listed in the reviews).

Response to Reviewers

Manuscript ID: OXFNSC-2025-008.R1
Title: Estradiol and Social Withdrawal in Addiction: A Sex-Specific Neuroendocrine Perspective

Response to the Editor

Dear Prof. Izquierdo,

I sincerely thank you and the reviewers for the thoughtful and constructive feedback. I appreciate the time and care taken to evaluate our manuscript. In response to the major revision request, I conducted a comprehensive, line-by-line revision of the manuscript.

Reviewer: 1

Comments to the Author
In this perspective, entitled “Estradiol and Social Withdrawal in Addiction: A Sex-Specific Neuroendocrine Perspective, authors provide an interesting and novel series of connections between addiction, social withdrawal, estradiol, and Black populations. Although this perspective is important and worthy of exploration, the key arguments of the perspective are, at best, loosely connected. More concerning however is the use of references that do not support the statements claimed in the paper. As such, although the paper has merit and warrants further investigation, it is currently not suitable for publication until the arguments posited are better connected and supported by appropriate references. Finally, future directions need to be better explained for the novelty of the work to shine.

Response: I thank the reviewer for this thoughtful assessment. I agree that the original draft required stronger conceptual integration, more precise citation support, and clearer future directions. In response, I restructured the manuscript to clarify the progression from reward-centric models to social withdrawal, estradiol modulation, and relapse vulnerability. I also conducted a systematic citation audit and revised the future directions to provide more concrete translational and methodological pathways.

Major concerns:
1. In the abstract, authors state multiple arguments that they set out to defend in the paper: 1) Social withdrawal is not just a consequence but a driver of relapse risk, 2) estradiol is a key biological regulator of social motivation/withdrawal, and 3) Estradiol-linked social effects are compounded by structural stressors that disproportionately burden marginalized populations.


The first argument regarding social withdrawal is perhaps the least novel, and accordingly has the most scientific support. Still, the references are not cited appropriately here. For example, “Social reward deficits function not merely as a consequence of drug use, but as a potentiating factor in addictive behavior” cites Venniro et al.’s seminal paper demonstrating that drug-exposed rats will prefer to operantly behave for a social reward over a drug reward. This paper does not support the notion that social reward deficits are a consequence of drug use and does not support the notion that social reward deficits are a potentiating factor in addictive behavior. This is one of many examples throughout the manuscript where a reference was selected without a close read of the findings.

Response: I appreciate this important critique. In response, I conducted a full review of all citations in the manuscript and replaced or removed references that did not directly support the claims made. I also revised the framing of social withdrawal to better reflect the literature, positioning it as a clinically meaningful vulnerability state that may contribute to relapse risk by interacting with stress and reward circuitry, rather than overstating the evidence. The abstract and main text were revised accordingly.


2. Regarding the second argument, authors are forceful in their linkage of estradiol to social motivation and reward. Although estradiol may be linked to social motivation, softening of this language may be appropriate for this perspective given the limited knowledge of the field. For example, authors claim that “…females also manifest heightened sensitivity to negative affective states during abstinence compared to males. Neuroendocrine factors, specifically 17B-estradiol, largely drive these sex-specific differences.” They then cite an influential review by Becker et al., which does indeed claim that females may have negative affective states during abstinence, but does not link this to estradiol and in fact, mainly uses animal studies when discussing a potential role of estradiol.


- Another example of this is found in section 3.2, where authors state “the manifestation of social withdrawal is distinctly reliant on neuroendocrine states” without a citation. They then state that stress responsivity, relapse vulnerability, and social interaction have sex differences, citing a 2010 Cahill review which focuses on emotional memory: “even stating sex hormones are crucial to the enhanced detail memory for emotional events in women, and in ways that remain almost wholly unexplored.” Given this citation, the claim that social withdrawal is reliant on neuroendocrine states, while likely true, is undersupported here.


- Further, limitations of the arugment are not discussed here. For example, although Brian Trainor, a pioneer in the field, has been cited, he has demonstrated that elements of social withdrawal are not estrogen-dependent (see Trainor et al., 2013, Hormones and Behavior). A limitations section would be balanced an appropriate.

Response: I thank the reviewer for this careful and constructive critique. I have revised the manuscript throughout to soften deterministic phrasing and now consistently describe estradiol as a modulatory factor rather than a singular driver of sex differences in social motivation, stress responsivity, or relapse-related behavior. In Section 3.2, I revised the language to clarify that hormonal context may influence the severity or persistence of withdrawal-related social disengagement, rather than determine it. I also added discussion of estrogen-independent mechanisms and now explicitly cite Trainor et al. (2013) to acknowledge that not all aspects of social stress responsivity are estrogen-dependent. These revisions were made to provide a more balanced and better supported perspective.


3. For the third argument, although critically necessary for future lines of research, it is perhaps not appropriate to state that in the abstract that the perspective will be discussing marginalized populations more generally when the discussion is specifically about Black individuals and Black women. One concern about stating “marginalized populations” is at worst, it can do a disservice to a specific marginalized population by suggesting that mechanisms of subjugation are generalized.


Here, authors are discussing structural racism and state “When these factors interface with hormonal vulnerabilities, they amplify susceptibility to SUDs and create pronounced inequities in access to comprehensive addiction care and support,” citing a 2011 paper by Algeria et al.. This paper does not discuss hormones or hormonal vulnerabilities, and so data would be necessary to make the likely accurate, but here unsupported, claim that systemic racism compounds with hormonal vulnerabilities.
Response: I appreciate this important point and agree that the language should be more precise. I revised the abstract and Section 4 to explicitly refer to Black women rather than marginalized populations more broadly. I also revised the discussion of structural stress and hormonal vulnerability to better distinguish what is directly supported by evidence from what is being proposed as a conceptual framework. In addition, I replaced unsupported citations and expanded Section 4.1 to provide a more mechanistically grounded explanation of how chronic structural stress and estradiol-sensitive circuits may converge. I now explicitly state that this interaction remains conceptual, while grounding it in evidence from stress neurobiology and endocrine modulation studies.


Other concerns
4. “animal models… often fail to capture the complexity of human clinical social presentations” is stated early in the manuscript, yet animals studies are quoted for the bulk of the perspective, typically without naming the model. Although this is true that animal models have limitations, their utility is not well described, and their contributions to the field are obfuscated. For example, in the section “Estradiol and Social Behavior Neurocircuitry,” every behavioral reference in this section refers to animal studies (refs 14-23) and yet rodents are not mentioned once. Throughout the manuscript, findings are often discussed without naming the model, and rodent and human studies are interspersed in a way that can be confusing or misleading.
Response: I agree and thank the reviewer for highlighting this issue. I revised the manuscript throughout to clearly distinguish between preclinical and human evidence. In Section 2, I now explicitly state that much of the mechanistic evidence derives from rodent models, and I identify model systems throughout with phrases such as “in rodent models” and “in human clinical populations.” I also revised the transitions between sections to better clarify the translational role and limitations of animal models.

Reviewer: 2

Comments to the Author
In this Brief Perspective, the authors present a relationship between the hormonal and societal influences on social withdrawal and affective neural circuitry driving drug addiction and relapse. This effort to bridge evidence from basic science studies and social epidemiology could be an important contribution to current conversations surrounding the need to better understand and address social determinants of health, both in clinical settings and when designing translational research experiments. The discussion of chronic stress and intersectional challenges faced by marginalized communities also brings up a factor that is inherently absent from preclinical studies but extremely relevant to the treatment of substance use conditions: the structural influences that many people experiencing addiction face, which can significantly impact a person’s ability to access, engage with, and maintain treatments. Overall, the manuscript provides a compelling conceptual bridge between neuroendocrine signaling and structural social determinants in addiction. However, the current draft suffers from significant citation inaccuracies and conceptual gaps that must be addressed before publication. Specifically, the linking evidence between these claims leaves much to be desired. Throughout the article, there are broad claims that lack sufficiently specific evidence. Linking mechanisms need more evidence, and actionable suggestions should be bolstered by direct steps towards implementation. While this perspective begins an important conversation, major revisions are necessary to make this suitable for publication. With revisions that more thoroughly and accurately cite evidence, this has the potential to add a valuable discussion of intersectional translational research in the field.

I thank the reviewer for this thoughtful and encouraging assessment. I appreciate the recognition of the manuscript’s conceptual contribution and agree that the original draft required stronger citation precision, better-supported linking mechanisms, and more actionable translational recommendations. In response, I thoroughly revised the manuscript to address each of these concerns.

Major Revisions
1. Sources need to be thoroughly reviewed. Several citations did not seem to fit the claim. To name a few:
- Section 2.1’s statement on dopamine synthesis, release and uptake inhibition cites [25] (Luine and Frankfurt, Neuroscience 2013), which doesn’t mention anything about dopamine. This source appears to be cited multiple other times in which it may not be appropriate, including section 2.3’s discussion of oxytocin.

- Section 2.3 claim about E2 strengthening the rewarding value of social interactions through increasing OTR expression also cites [38] (Trainor et al, Hormones and Behavior 2008), which is about photoperiod and doesn’t mention oxytocin or reward.

- Section 4 introduces the Weathering Hypothesis and discussion of intersectional structural considerations extremely vaguely and makes little attempt to provide evidence for the “bio-social vulnerability cascade.”

Response: I appreciate this careful review of the references. In response, I conducted a comprehensive citation audit throughout the manuscript and corrected instances where sources did not directly support the associated claims. In Section 2.1 and Section 2.3, I replaced mismatched citations with more appropriate sources or revised the text to better reflect the available evidence. I also substantially revised Section 4 to move beyond vague conceptual language and provide a more evidence-based discussion of the Weathering Hypothesis, allostatic load, and the proposed bio-social vulnerability cascade.

2. Section 4 could be improved by further discussion of community-engaged research methodologies: provide examples of successful studies or how current models could be improved, rather than just stating that these should be implemented. Also, provide specific evidence for the mechanistic cascade; i.e., how exactly does high allostatic load interact with E2-sensitive circuits like the mPFC or LHb to accelerate the loop?

Response: I thank the reviewer for this helpful suggestion. In response, I expanded Section 4.1 to provide greater mechanistic grounding, including discussion of chronic HPA axis activation, sustained glucocorticoid exposure, altered inflammatory signaling, and their effects on synaptic plasticity in stress-sensitive regions such as the mPFC, amygdala, and LHb. I also revised Section 4.2 to include specific examples of community-engaged and translational research approaches, including community advisory boards, peer-recovery specialists, and digital social-support tools embedded within hormone-informed longitudinal studies.

3. Similarly, Section 5 introduces “precision addiction medicine” and “targeted hormone modulation” within one brief paragraph, overall contains limited sources, and does not seem sufficient to convince a clinical or basic science audience that these methodologies would be effective. Suggest that authors provide concrete examples of community-engaged research methodologies rather than just stating they should be used.

Source [95] (Yamac and Bakir, Rev Assoc Med Bras 1992; 2025) should also be reviewed, as it is used in a statement about targeted hormone modulation but seems irrelevant.
Response: I appreciate this critique and agree that the earlier framing was too compressed and potentially overstated. I revised Section 5 to emphasize that the immediate next step is observational and longitudinal research, not direct hormone-based intervention. I now describe precision addiction medicine in this context as the integration of endocrine biomarkers, cumulative stress measures, and social network metrics into predictive models of relapse vulnerability, pending rigorous validation. I also removed or replaced citations that did not directly support these claims, including the source noted by the reviewer. In addition, I strengthened the section by adding more concrete examples of how social-support interventions could be timed during hormonally sensitive windows in future studies.

Minor Revisions

4. Section 3.1 clearly delineates evidence from rodent SUD models versus human clinical evidence. This approach should be taken more frequently, because in other sections it was at times unclear whether behaviors/neuroendocrine patterns described human or animal studies.
Response: I thank the reviewer for this observation. I revised the manuscript throughout to more consistently distinguish preclinical from human evidence, following the structure used in Section 3.1.

5. In line 14-15 the use of 4“cruel” to describe this paradoxical phenomenon introduces a personal tone that feels less objective than the rest of the perspective.

Response: I agree and have removed this wording to maintain a more objective tone.

6. The recommendation for "mandatory reporting standards" in Section 5.1 is a strength but should explicitly mention the importance of reporting both sex and gender variables to align with the intersectional goals of the paper.

Response: I appreciate this important suggestion. In response, I revised Section 5.1 to explicitly distinguish biological sex and self-identified gender and to recommend transparent reporting of both variables in future addiction research.

I are grateful for the reviewers’ thoughtful and constructive feedback. These revisions have substantially strengthened the clarity, evidentiary rigor, and translational grounding of the manuscript. I hope the revised version addresses all concerns and contributes meaningfully to ongoing discussions at the intersection of addiction neuroscience, neuroendocrine modulation, and structural determinants of health.

Sincerely,

**Round 2**

16-May-2026

Dear Dr. Kimble,

Manuscript ID OXFNSC-2025-008.R1 entitled "Estradiol and Social Withdrawal in Addiction: A Sex-Specific Neuroendocrine Perspective" which you submitted to the Oxford Open Neuroscience, has been reviewed.  The comments of the reviewers are included at the bottom of this letter.

Please note that this journal operates with transparent peer review. This means that if your submission is accepted for publication, the full peer review history of your article will publish online alongside your article. This includes reviewer comments, editor decision letters, and your author responses.

The reviewers have recommended publication, but also suggest some minor revisions to your manuscript.  Therefore, I invite you to respond to the reviewers' comments and revise your manuscript within the next 30 days from todays date.

To revise your manuscript, log into https://mc.manuscriptcentral.com/oxfnsc and enter your Author Centre, where you will find your manuscript title listed under "Manuscripts with Decisions."  Under "Actions," click on "Create a Revision."  Your manuscript number has been appended to denote a revision.

You may also click the below link to start the revision process (or continue the process if you have already started your revision) for your manuscript. If you use the below link you will not be required to login to ScholarOne Manuscripts.

*** PLEASE NOTE: This is a two-step process. After clicking on the link, you will be directed to a webpage to confirm. ***

https://mc.manuscriptcentral.com/oxfnsc?URL_MASK=7261d0f3cdbe450a8ecfd8782c2d5529

You will be unable to make your revisions on the originally submitted version of the manuscript.  Instead, revise your manuscript using a word processing program and save it on your computer.  Please also highlight the changes to your manuscript within the document by using the track changes mode in MS Word or by using bold or colored text.

<strong>REMINDER:</strong> if you have not already done so, when submitting your revised manuscript, please include a short alternative-text description under the figure legend for each figure. Alt-text is required to ensure the accessibility of our publications. Guidance is available on the journal’s Author Guidelines webpage and at <a href="https://academic.oup.com/pages/for-authors/journals/preparing-and-submitting-your-manuscript" target="_new">https://academic.oup.com/pages/for-authors/journals/preparing-and-submitting-your-manuscript</a>

Once the revised manuscript is prepared, you can upload it and submit it through your Author Centre.

When submitting your revised manuscript, you will be able to respond to the comments made by the reviewer(s) in the space provided.  You can use this space to document any changes you make to the original manuscript.  In order to expedite the processing of the revised manuscript, please be as specific as possible in your response to the reviewer(s).

IMPORTANT:  Your original files are available to you when you upload your revised manuscript.  Please delete any redundant files before completing the submission.

Because we are trying to facilitate timely publication of manuscripts submitted to the Oxford Open Neuroscience, your revised manuscript should be uploaded within the next 30 days from todays date.  If it is not possible for you to submit your revision in a reasonable amount of time, we may have to consider your paper as a new submission.

Once again, thank you for submitting your manuscript to the Oxford Open Neuroscience and I look forward to receiving your revision.

Sincerely,
Prof. Alicia Izquierdo
Senior Editor, Oxford Open Neuroscience
aizquie@psych.ucla.edu


Reviewer: 1

Comments to the Author
It was a pleasure to review this perspective on E2 signaling, social withdrawal, and addiction. I particularly believe the sections on intersectionality and the “call to action” have been robustly improved and given the nuance they deserve, and I applaud the authors for this. I still think that the preclinical neuroscientific section requires some more concrete findings and evidence to better serve the readership of the journal, specifically when it comes to vaguely discussing associations between behavior and hormonal signaling described below.
Major Concerns
- Several times throughout the manuscript verbs like “modulate,” “alter,” “influence,” “interacts with”, “regulate,” and “mediate” (E.g., Page 2, 40-46; Page 3, 25-26; Page 4, 26-27). These are neutral verbs that do not inform the reader of directionality of the effect (meaning that they must go to the reference to learn how E2/hormones affect social behavior. As this is review of that literature, please include directionality to ease readability.
o For example, on Page 4, 31-33: “In rodent models, ovarian hormone depletion can exacerbate stress-related behaviors and alter social interaction patterns, while hormone replacement can partially reverse some of these effects”
♣ Here, I do not know what exacerbate entails, what behaviors are being tested, what alters means, or how social interaction patterns are changing. This makes the statement about hormone replacement less effective, because I do not know the directionality and therefore cannot know what is being reversed.
o
- Similarly, several times throughout the manuscript it is noted that brain regions affected by stress “overlap” with those affected by E2 (Page 1, 54-55; Page 2, 50-51; Page 4, 45-48) or via “shared” systems (Page 3 17-18; Page 4 10-11; Page 5, 30, 38-39). A brain region can have multiple functions, and correlation here, despite the use of the word “may,” dose not imply causation. E2 and stress likely interact with most, if not all regions of the brain in some regard, so I worry about the specificity of the arguments being made here.

Minor Concerns
- Page 1, 50-52: “Social withdrawal, characterized by reduced engagement with social reinforcers and diminished motivation for affiliative interaction, commonly emerges during substance use and abstinence.” as well as Page 3, 46-48 “Social withdrawal, defined as reduced social interaction, diminished responsiveness to affiliative cues, and avoidance of social contexts, is frequently observed during substance use and abstinence.”
o Social withdrawal is a complicated phenotype that has several derivatives and likely follows a “many paths up the mountain” concept. Some individuals who have been exposed to cocaine find social interactions less rewarding (i.e., social anhedonia), but others may be embarrassed by their behavior while they were using or disrupted their relationships with consequences of their use (but importantly, are still equally interested in social interaction). Further, some individuals might feel like they cannot interact with those who do not understand their experience, or that they cannot interact with their previous social supports if those people are still using substances. Thus, we cannot presume why a given individual is withdrawing socially – social withdrawal may be defined as reduced interaction, but whether it is due to reduced affliation or avoidance (or a combination of both) is likely individualized clinically.
- Although later in the manuscript detail is given to the differentiation between sex and gender, and the importance of comparing males + estradiol or ovariectomized females in preclinical studies, this would be pertinent to bring up earlier in the manuscript – as it currently stands, estradiol feels equated with female sex during the neurobiological sections, which could be perceived reductive.
- Page 3, 36-37 + Page 4, 36-37: Same point about “broader neuroendocrine context/network” made twice
- Page 3, 9-11: LHb acronym introduced twice
- Page 3, 15-16: Use LHb acronym if introduced
- Figure 1b: tenses are not consistent


Reviewer: 2

Comments to the Author
In this response, the author presented a clear narrative linking stress and hormone changes with social behavior patterns during addiction and relapse. There was significant improvement from the original draft in the use of robust, specific evidence to support claims throughout the manuscript, especially in the neuroendocrine and bio-social sections. I especially appreciated the changes in Section 3 and 5, where the added level of evidence and nuance painted a clearer picture of the how social withdrawal serves as a stress-amplifying state that accelerates the route to addiction, and more specific suggestions were made for how the field could implement these considerations into research frameworks. Throughout the narrative, evidence from preclinical and clinical studies was clearly identified and well-balanced, giving readers a broad translational view of how estradiol may moderate these stress-influenced states during addiction and relapse. Based on the sources cited, I still think the estradiol-driven mechanism at play could be described slightly more because the neurobiological evidence included in the manuscript highlights the same core overarching points (HPA axis hyperactivity, hormone-driven changes), and the breakdown of this process is relatively brief (see minor revision 2). Finally, with Section 4’s discussion on intersectional considerations being a core argument that sets this review apart from much translational work in the field, I feel that a few minor edits could tighten the narrative and maximize the impact this section has on readers (see minor revision 3). Overall, this review opens up an important discussion on the connections between structural stressors, sex and gender, social behavior, and addiction, and with a few minor revisions I think it would be a valuable conversation to bring to the field.

Minor Revisions
1) There are two instances where nearly identical phrasing from earlier in the narrative is repeated. Both of these are core claims by the paper, but in a perspective of this length it felt like making each claim twice weakened the impact, as if it could not be restated in different words or elaborated upon.
a) Section 1 “Thus, rather than functioning solely as a downstream consequence of substance use, social withdrawal may contribute to relapse risk” feels repeated in Section 3 “Rather than functioning solely as a downstream behavioral consequence of drug exposure… influence relapse risk”  
b) Section 2.2 “not all components of stress reactivity are estrogen-dependent, underscoring that E2 acts as a neuromodulator rather than” feels repeated in Section 3.2 “However, not all aspects of social stress responsivity are estrogen-dependent, and evidence indicates that multiple neuromodulator systems interact…”
2) There are a few instances where the authors could add a citation (likely from sources already included) or otherwise elaborate to drive home essential mechanistic points:
a) In 2.1, “genomic and rapid membrane-initiated estrogen receptor signaling” is mentioned briefly, and this feels like an opportunity to expand on the molecular mechanisms at play. Perhaps breaking down each of these types of signaling control would serve a dual purpose of further convincing basic scientists of the claim, and providing additional context for readers who may be less familiar with genomic mechanisms (could probably cite existing reference 21, Zheng et al., Translational Psychiatry 2018 here).
b) In 2.2, elaborating on the hormone-sensitive plasticity referenced in paragraph 1 could strengthen the claim in paragraph 2 that mentions how fluctuations in hormonal state may influence stress-induced adaptations.
3) The section on structural stress and intersectionality is much improved, with strong evidence for the weathering hypothesis and discussion of persistent HPA axis activation. In Section 4.2, there are just a few “buzz-words” that feel like they are one degree of separation away from the evidence provided, and would be stronger with supporting examples.
a) “…culturally-responsive care strategies” -- while hormone-informed studies (targeting care during hormonally sensitive periods, tracking reproductive transitions) and community-engaged studies (incorporating peer recovery specialists, community advisory boards) are mentioned, the provided thus far does not seem to reference examples what “culturally-informed” care means or how this would incorporate these factors. I think this is a point many people would agree with, and would love to see an example of how to address culture in scientific research.
b) “…move beyond reductionist frameworks” -- while this section adds valuable considerations to current research frameworks, it does not explicitly highlight how current frameworks are “reductionist.” It feels there is room for speculation on the extent to which current frameworks lack these considerations, since many different factors have been mentioned.
i) Is it a callback to expanding beyond animal models that simplify the social complexity of the human experience?
ii) Is it broadly referencing intersectional considerations and suggesting to move beyond clinical practices that focus on symptom management to integrating longer-term risk factors?
iii) Is it specifically referencing this paragraph on designing frameworks that center Black women and other marginalized communities experiencing greater bio-social vulnerability due to structural stress?

Associate Editor
Comments to the Author:

Dear Author,
Thank you for your work on the manuscript. Please see reviewers comments for minor revisions.

Response to Reviewers
Manuscript ID: OXFNSC-2025-008.R1
Title: Estradiol and Social Withdrawal in Addiction: A Sex-Specific Neuroendocrine Perspective

Response to the Editor
Dear Prof. Izquierdo,

I sincerely thank you and the reviewers for the thoughtful and constructive feedback. I appreciate the time and care taken to evaluate our revised manuscript. In response to the minor revision request, I conducted a careful revision of the manuscript to clarify the directionality of estradiol-related effects, refine language around overlapping stress- and hormone-sensitive circuits, strengthen mechanistic detail, and further clarify the social and structural context of the proposed framework. I also added figure alt text in accordance with the journal’s accessibility requirement.

Reviewer: 1
Comments to the Author
It was a pleasure to review this perspective on E2 signaling, social withdrawal, and addiction. I particularly believe the sections on intersectionality and the “call to action” have been robustly improved and given the nuance they deserve, and I applaud the authors for this. I still think that the preclinical neuroscientific section requires some more concrete findings and evidence to better serve the readership of the journal, specifically when it comes to vaguely discussing associations between behavior and hormonal signaling described below.
Response: I thank the reviewer for this thoughtful and encouraging assessment. I appreciate the recognition that the sections on intersectionality and the call to action have improved. I agree that the preclinical neuroscience sections would benefit from more concrete language and clearer directionality. In response, I revised Sections 2 and 3 to specify the direction of estradiol-related effects where supported by the cited literature, to better distinguish preclinical from human evidence, and to avoid overinterpreting shared or overlapping circuit involvement as evidence of causality.

Major concerns:
1. Several times throughout the manuscript verbs like “modulate”, “alter”, “influence”, “interacts with”, “regulate”, and “mediate” are used without specifying directionality. The reviewer requested more concrete descriptions of how E2/hormones affect social behavior and stress-related outcomes.
Response: I thank the reviewer for this important point. I agree that the prior wording was sometimes too general and required readers to consult the cited sources to determine the direction of the effect. I revised Sections 2.1, 2.2, 2.3, and 3.2 to include more directional language where the literature supports it. For example, I now describe E2 as enhancing dopamine release and altering dopamine receptor sensitivity in the striatum; estrogen receptor signaling as increasing oxytocin receptor expression in specific brain regions; low-E2 states as being associated in some models with reduced social recognition, altered aggression, or decreased affiliative-related behavior; and ovarian hormone depletion as being associated in some paradigms with increased anxiety- or avoidance-like behavior and reduced social investigation. These revisions provide more concrete evidence for readers and clarify the directionality of hormonal effects without overstating uniformity across models.

2. Several times throughout the manuscript it is noted that brain regions affected by stress “overlap” with those affected by E2 or via “shared” systems. The reviewer noted that a brain region can have multiple functions and that correlation, despite the use of “may,” does not imply causation. The reviewer expressed concern about the specificity of the arguments being made.
Response: I agree with the reviewer and revised the manuscript to avoid implying that anatomical or circuit overlap establishes causality. I replaced broad references to “overlap” and “shared systems” with more precise language, including “converge on specific stress- and affect-regulatory nodes,” “partially overlapping mesolimbic circuits,” and “candidate pathways.” I also added explicit caution that this convergence does not itself establish causality. These revisions clarify that the manuscript proposes a conceptual and testable framework rather than asserting causality from circuit overlap alone.

Other concerns
3. The reviewer noted that social withdrawal is a complicated phenotype with several derivatives and likely follows a “many paths up the mountain” concept. The reviewer cautioned that social withdrawal should not be presumed to reflect reduced affiliation or avoidance alone, because it may reflect shame, disrupted relationships, stigma, inability to interact with those who do not understand the experience, or avoidance of previous substance-using social supports.
Response: I thank the reviewer for this important clarification. I revised the Introduction and Section 3 to define social withdrawal more carefully as a heterogeneous phenotype. The manuscript now distinguishes the observable behavior of reduced social engagement from the multiple possible pathways that may produce it. Specifically, I clarified that social withdrawal may reflect social anhedonia, shame or stigma, disrupted relationships, avoidance of substance-associated social networks, reduced access to supportive non-using peers, or the perception that others cannot understand the recovery experience. I also revised Section 3 to reinforce that observable reductions in social contact may arise from multiple pathways rather than a single underlying mechanism.

4. Although later in the manuscript detail is given to the differentiation between sex and gender, and the importance of comparing males plus estradiol or ovariectomized females in preclinical studies, this would be pertinent to bring up earlier in the manuscript. As it currently stands, estradiol feels equated with female sex during the neurobiological sections, which could be perceived as reductive.
Response: I agree and added an early clarification in the Introduction immediately after estradiol is introduced. This revision makes clear that estradiol should not be treated as equivalent to female sex and that sex, gender, and hormonal state are related but distinct variables. This addition brings the sex/gender/hormonal-state distinction forward before the main neurobiological sections.

5. Page 3, 36–37 and Page 4, 36–37 make the same point about “broader neuroendocrine context/network” twice.
Response: I revised this wording to reduce repetition. In Section 2.3, I retained the concept that estradiol acts within a multi-system neuroendocrine context. In Section 3.2, I replaced the repeated wording with a more specific behavioral prediction: that hormonal context may influence the severity, persistence, or recovery of withdrawal-related social disengagement, particularly when abstinence-related stress and reduced social reinforcement occur together.

6. Page 3, 9–11: LHb acronym introduced twice.
Response: I corrected this. LHb is now introduced once in Section 2.2 and used consistently thereafter.

7. Page 3, 15–16: Use LHb acronym if introduced.
Response: I revised the manuscript to use “LHb” consistently after the first definition.

8. Figure 1b: tenses are not consistent.
Response: I revised the Figure 1 legend to use consistent present tense throughout. I also corrected the Panel B description so that the figure description remains grammatically consistent and uses present-tense phrasing.

Reviewer: 2
Comments to the Author
In this response, the author presented a clear narrative linking stress and hormone changes with social behavior patterns during addiction and relapse. There was significant improvement from the original draft in the use of robust, specific evidence to support claims throughout the manuscript, especially in the neuroendocrine and bio-social sections. I especially appreciated the changes in Section 3 and 5, where the added level of evidence and nuance painted a clearer picture of how social withdrawal serves as a stress-amplifying state that accelerates the route to addiction, and more specific suggestions were made for how the field could implement these considerations into research frameworks. Throughout the narrative, evidence from preclinical and clinical studies was clearly identified and well-balanced, giving readers a broad translational view of how estradiol may moderate these stress-influenced states during addiction and relapse. Based on the sources cited, I still think the estradiol-driven mechanism at play could be described slightly more because the neurobiological evidence included in the manuscript highlights the same core overarching points, and the breakdown of this process is relatively brief. Finally, with Section 4’s discussion on intersectional considerations being a core argument that sets this review apart from much translational work in the field, I feel that a few minor edits could tighten the narrative and maximize the impact this section has on readers.
Response: I thank the reviewer for this thoughtful and constructive assessment. I appreciate the recognition that the revised manuscript presents a clearer translational narrative and better distinguishes preclinical and clinical evidence. I agree that the estradiol-related mechanisms could be described in greater detail and that Section 4 would benefit from more concrete examples and tighter wording. In response, I expanded the discussion of estrogen receptor signaling and hormone-sensitive plasticity, reduced repeated phrasing, clarified the meaning of culturally responsive care, and specified what is meant by moving beyond reductionist frameworks.

Minor Revisions
1. Section 1 “Thus, rather than functioning solely as a downstream consequence of substance use, social withdrawal may contribute to relapse risk” feels repeated in Section 3 “Rather than functioning solely as a downstream behavioral consequence of drug exposure… influence relapse risk.”
Response: I agree and revised Section 3 so that it no longer repeats the Introduction’s framing. Section 3 now focuses on social withdrawal as a behavioral state linking abstinence-related negative affect, reduced social reinforcement, and stress-sensitive drug seeking. This revision preserves the central argument while reducing repetitive phrasing.

2. Section 2.2 “not all components of stress reactivity are estrogen-dependent, underscoring that E2 acts as a neuromodulator rather than…” feels repeated in Section 3.2 “However, not all aspects of social stress responsivity are estrogen-dependent…”
Response: I revised Section 3.2 to avoid repeating the same phrasing. Section 2.2 retains the mechanistic caveat, while Section 3.2 now uses the evidence to generate a more specific behavioral prediction. The revised section now clarifies that findings are not consistent across all models, that stress-induced social withdrawal can occur independently of adult gonadal hormones in some paradigms, and that non-estrogenic systems also contribute to social withdrawal phenotypes. This preserves the nuance while avoiding near-duplicate wording.

3. In 2.1, “genomic and rapid membrane-initiated estrogen receptor signaling” is mentioned briefly, and this feels like an opportunity to expand on the molecular mechanisms at play. Perhaps breaking down each of these types of signaling control would serve a dual purpose of further convincing basic scientists of the claim and providing additional context for readers less familiar with genomic mechanisms. The reviewer suggested that existing reference 21, Zhang et al., Translational Psychiatry 2018, could be cited here.
Response: I agree and expanded Section 2.1 to briefly distinguish genomic estrogen receptor signaling from rapid membrane-initiated estrogen receptor signaling. I now explain that genomic estrogen receptor signaling can alter transcriptional programs that shape receptor expression, synaptic organization, and longer-timescale changes in neuronal excitability, whereas membrane-initiated estrogen receptor signaling can more rapidly engage intracellular kinase cascades and local synaptic mechanisms. I also cite the existing Zhang et al. reference where appropriate.

4. In 2.2, elaborating on the hormone-sensitive plasticity referenced in paragraph 1 could strengthen the claim in paragraph 2 that mentions how fluctuations in hormonal state may influence stress-induced adaptations.
Response: I agree and added more detail in Section 2.2 to clarify what is meant by hormone-sensitive plasticity and how it could shape stress-induced neural adaptations. I now describe hormone-sensitive plasticity as potentially including estrous- or reproductive-state-dependent changes in inhibitory tone, synaptic strength, receptor expression, and functional connectivity within stress- and affect-regulatory circuits. I also clarified that the same stressor may produce different neural and behavioral consequences depending on hormonal state.

5. In Section 4.2, “culturally-responsive care strategies” feels one degree removed from the evidence provided. The reviewer requested examples of what culturally informed care means or how culture would be incorporated into scientific research.
Response: I thank the reviewer for this helpful suggestion. I revised Section 4.2 to define culturally responsive approaches more concretely and added two new references to support this point. The revised text now gives examples of how culturally responsive care can be operationalized in addiction research and treatment, including community advisory boards, peer-recovery specialists, culturally adapted intervention materials, attention to participants’ explanatory models of substance use and recovery, and trusted community settings. I also added the Imani Breakthrough project as a concrete example of a culturally centered, community-driven opioid recovery initiative.

6. The reviewer asked the authors to clarify what they mean by “move beyond reductionist frameworks,” including whether this refers to expanding beyond animal models, moving beyond symptom management, or centering marginalized communities experiencing greater bio-social vulnerability due to structural stress.
Response: I agree and revised the final sentence of Section 4.2 to explicitly define what is meant by reductionist frameworks in this manuscript. The revision clarifies that the phrase refers to frameworks that isolate hormones, drug reward, or individual symptoms from the broader structural, gendered, and social contexts in which relapse vulnerability develops.

Additional Editorial Revision
The decision letter reminded the author to include a short alternative-text description under the figure legend for each figure.
Response: I added alt text under the Figure 1 legend in accordance with the journal’s accessibility requirement.

I am grateful for the reviewers’ thoughtful and constructive feedback. These revisions have strengthened the manuscript’s precision, mechanistic clarity, and translational relevance. I hope the revised version addresses all remaining concerns.

Sincerely,
